# A scoping review on women’s sexual health in the postpartum period: opportunities for research and practice within low-and middle-income countries

**DOI:** 10.1186/s12978-022-01399-6

**Published:** 2022-05-08

**Authors:** Shannon N. Wood, Alexandria Pigott, Haley L. Thomas, Chloe Wood, Linnea A. Zimmerman

**Affiliations:** 1 Department of Population, Family and Reproductive Health, Johns Hopkins Bloomberg School of Public Health, Baltimore, MD USA; 2 Department of Obstetrics and Gynecology, Creighton University School of Medicine Phoenix, Phoenix, AZ USA

**Keywords:** Sexual health, Postpartum, Sexual function, Sexual dysfunction

## Abstract

**Background:**

Women’s sexual health is generally defined and explored solely in relation to reproductive capacity, and often omits elements of sexual function and/or dysfunction. Concerted focus is given to women’s health during pregnancy; however, women’s sexual health is largely neglected after childbirth. This scoping review explored how the sexual health of postpartum women has been defined, measured, and researched in low- and middle-income countries (LMICs).

**Methods:**

Articles eligible for review were those that investigated women’s sexual health during the first 12 months postpartum and were conducted among women aged 15–49 in LMICs. Eligibility was further restricted to studies that were published within the last 20 years (2001–2021). The initial PubMed search identified 812 articles, but upon further eligibility review, 97 remained. At this time, the decision was made to focus this review only on articles addressing sexual function and/or dysfunction, which yielded 46 articles. Key article characteristics were described and analyzed by outcome.

**Results:**

Of the final included articles, five studies focused on positive sexual health, 13 on negative sexual health, and the remaining 28 on both positive and negative sexual health or without specified directionality. The most common outcome examined was resumption of sex after childbirth. Most studies occurred within sub-Saharan Africa (n = 27), with geographic spread throughout the Middle East (n = 10), Asia (n = 5), North Africa (n = 3), and cross-geography (n = 1); notably, all five studies on positive sexual health were conducted in Iran. Negative sexual health outcomes included vaginismus, dyspareunia, episiotomy, perineal tears, prolapse, infection, obstetric fistula, female genital cutting, postnatal pain, uterine prolapse, coercion to resume sex, sexual violence, and loss of sexual desire/arousal. Most studies were quantitative, though eight qualitative studies elucidated the difficulties women endured in receiving information specific to sexual health and hesitance in seeking help for sexual morbidities in the postpartum period.

**Conclusions:**

Overall, the evidence base surrounding women’s sexual health in the postpartum period within LMICs remains limited, with most studies focusing solely on the timing of resumption of sex. Integration of sexual health counseling into postnatal care and nonjudgmental service provision can help women navigate these bodily changes and ultimately improve their sexual health.

**Supplementary Information:**

The online version contains supplementary material available at 10.1186/s12978-022-01399-6.

## Plain language summary

Women’s sexual health is often studied in relation to reproductive health and childbearing. While reproductive health during pregnancy and immediately after is well documented, it remains unclear how women’s sexual health is addressed, particularly within low- and middle-income countries. The aim of this review is to understand how researchers have measured, defined, and examined postpartum sexual health. In October 2021, we searched PubMed database with the following criteria: published in the last 20 years; conducted in a low- or middle-income context; examined sexual function and/or dysfunction among women aged 15–49 within 1 year after childbirth. From this inclusion criteria, we identified 46 relevant articles. Most studies were conducted in sub-Saharan Africa. Only five studies focused exclusively on positive sexual health, and the majority of studies examined the resumption of sex after childbirth. Multiple qualitative studies described women’s reluctance to seek help for postpartum sexual health issues and highlighted the difficulties they faced in receiving information specific to sexual health. Overall, the evidence base surrounding women’s sexual health after childbirth within low- and middle-income contexts is limited. Future research should examine sexual health beyond resumption of sex after childbirth and explore barriers to help-seeking for women experiencing sexual health issues. Further exploration of positive sexual health is needed across contexts.

**Supplementary Information:**

The online version contains supplementary material available at 10.1186/s12978-022-01399-6.

## Introduction

Women’s health is often framed in relation to reproductive capacity, with less attention on the wide spectrum of health outcomes that are both unique to women or disproportionately impact women [1, 2]. The sexual and reproductive health (SRH) field has been largely criticized for underemphasis on the “S” in SRH, where these critiques highlight underinvestment in and lack of policies surrounding women’s sexual health [3, 4]. Sexual health, or “the positive and respectful approach to sexual relationships, including pleasurable and safe sexual experiences, free from coercion, discrimination, or violence” [5], is a cornerstone of Sustainable Development Goal-5 [6], however, indicators notably center around infringements on sexual rights, including sexual violence, child marriage, and female genital cutting (FGC), rather than addressing positive outcomes, such as voluntary resumption of sex or sexual pleasure.

Pregnancy and childbirth are profound physical and psychological transition periods for women, and vast literature has documented the impact of pregnancy on women’s sexual health, including the necessity of sexual health counseling during antenatal care visits and childbirth classes [7, 8]. Further, a number of studies from high-income countries, including systematic reviews, have explored specific sexual function or dysfunction outcomes during the postpartum period [9–11]. These studies reveal the high prevalence of sexual morbidities, such as dyspareunia, incontinence, lack of desire, and change in sensations of pleasures following childbirth, as well need for effective interventions and counseling strategies to navigate them [9–11]. Additionally, the effects of psychological changes within the postpartum period must be considered, as postnatal depression affects as many as one in five women and can have a profound impact on sexual desire and the perception of sexual pleasure [7]. Despite the research supporting interventions, postpartum sexual health care remains regionalized, under-funded, and without policy support, even within high-income countries.

In low- and middle-income countries (LMICs), many women deliver at home or in facilities that are ill-equipped for obstetric emergencies [12], placing them at increased risk of both short- and long-term delivery complications [13, 14], including sexual health morbidities [15]. Antenatal and postnatal care are key intervention points for sexual health education and counseling; however, coverage of these services varies substantially, both within and across LMIC contexts [7, 16, 17] Moreover, recent efforts to estimate the coverage of quality services have found substantial gaps [16, 17]. Postnatal care, which consistently has lower coverage than antenatal care [18, 19], focuses largely on life-threatening danger signs to the child, with maternal health often limited to counseling on postpartum contraceptive use to prevent short interval pregnancies. Given these gaps in care, much less is known about the prevalence of postpartum sexual health complications and/or practices to mitigate dysfunctions and promote positive sexual health within LMICs.

Against this backdrop, the present study aims to synthesize the current literature on women’s sexual health in the postpartum period, with a focus on research specific to sexual function and dysfunction among women living in LMICs. Findings can inform research and practice guidelines within LMICs.

## Methods

### Scoping review procedures

A scoping review was conducted to identify and summarize studies from LMICs, as described by Arksey and Malley [20], Levac, Colquhoun and O’Brien [21], and the PRISMA Extension for Scoping Reviews [22]. First, a search strategy was developed with the assistance of library informationists (Additional file 1: Table S1). This search was conducted within PubMed in October 26, 2021, with identified references imported into Covidence, a systematic literature review management program. Titles and abstracts of each reference were screened by one Masters-level researcher, with questions flagged for the first author. Full-text articles were then reviewed by two Masters-level researchers to determine final eligibility. Throughout the review process, eligibility criteria were discussed with the entire authorship team and any disputes resolved via discussion. All decisions were documented within Covidence software.

Studies were eligible for inclusion if they were peer-reviewed, English-language articles, published within the past twenty years (2001 to 2021), and explored women’s sexual health in the postpartum period. Initially, the team adopted a broad definition of sexual health—in line with the World Health Organization’s definition and examples—which included wide-ranging topics such as sexual expression, relationships, pleasure, sexually transmitted infections (STIs), sexual dysfunction, sexual violence, and harmful sexual practices [23]. Given the vast, well-documented literature on postpartum contraception, family planning, and return to fertility, the team chose to not include these terms within the search. During the full-text review of the literature, the team identified 97 articles that met the initial review criteria; of note, this initial review included topics such as human immunodeficiency virus (HIV), sexually transmitted infections (STIs), reproductive cancers, fistula, reproductive tract infections (RTIs), uterine prolapse, FGC, sexual violence, sexual function, and sexual dysfunction. To allow for a more focused discussion while ensuring comprehensive coverage of positive and negative sexual health outcomes, the research team chose to narrow the inclusion criteria and focus solely on sexual function and/or dysfunction (defined further below); many of the initial topics were also included if the article concurrently discussed sexual function/dysfunction. Future work aims to explore the myriad of additional sexual health needs of women in the postpartum period.

*Final inclusion criteria:*

- Studies that examined women’s sexual health during the postpartum period.
  - For the purpose of this scoping review, the postpartum period was defined as one year after delivery.
  - To allow for a comprehensive lens of sexual health and be in line with the World Health Organization's definition, sexual health included both negative and positive experiences; this review focuses on sexual function (including resumption of sexual activity, sexual pleasure, sexual satisfaction, arousal, intimacy, orgasm) and sexual dysfunction (including dyspareunia, sexual violence, sexual coercion)
- Studies focused on women of childbearing age (ages 15–49)
- Studies published in English
- Studies conducted in low or low-middle economies, as defined by the World Bank lending groups (2021–2022 classification)
- Studies published within the last 20 years (between 2001 and 2021)

*Final exclusion criteria*

- Studies that were not conducted with women less than one year postpartum (i.e., studies with only male partners or service providers)
- Studies not specific to sexual health, and specifically sexual function/dysfunction, as defined within the inclusion criteria
- Studies specific to rapid repeat pregnancy, unintended pregnancy, abortion, postpartum family planning, contraceptive side-effects, or postpartum fertility, including studies that examined sexual abstinence only in relation to fertility and family planning
- Literature reviews, case reports, study protocols, and grey literature
- Studies focused on morbidities experienced before or after, rather than during, the postpartum period
- Studies that focused on the perspectives of men and men’s sexual activity within the postpartum period
- Studies that focus on highly specific subgroups, such as HIV positive women only

### Data extraction

**Fig. 1:**
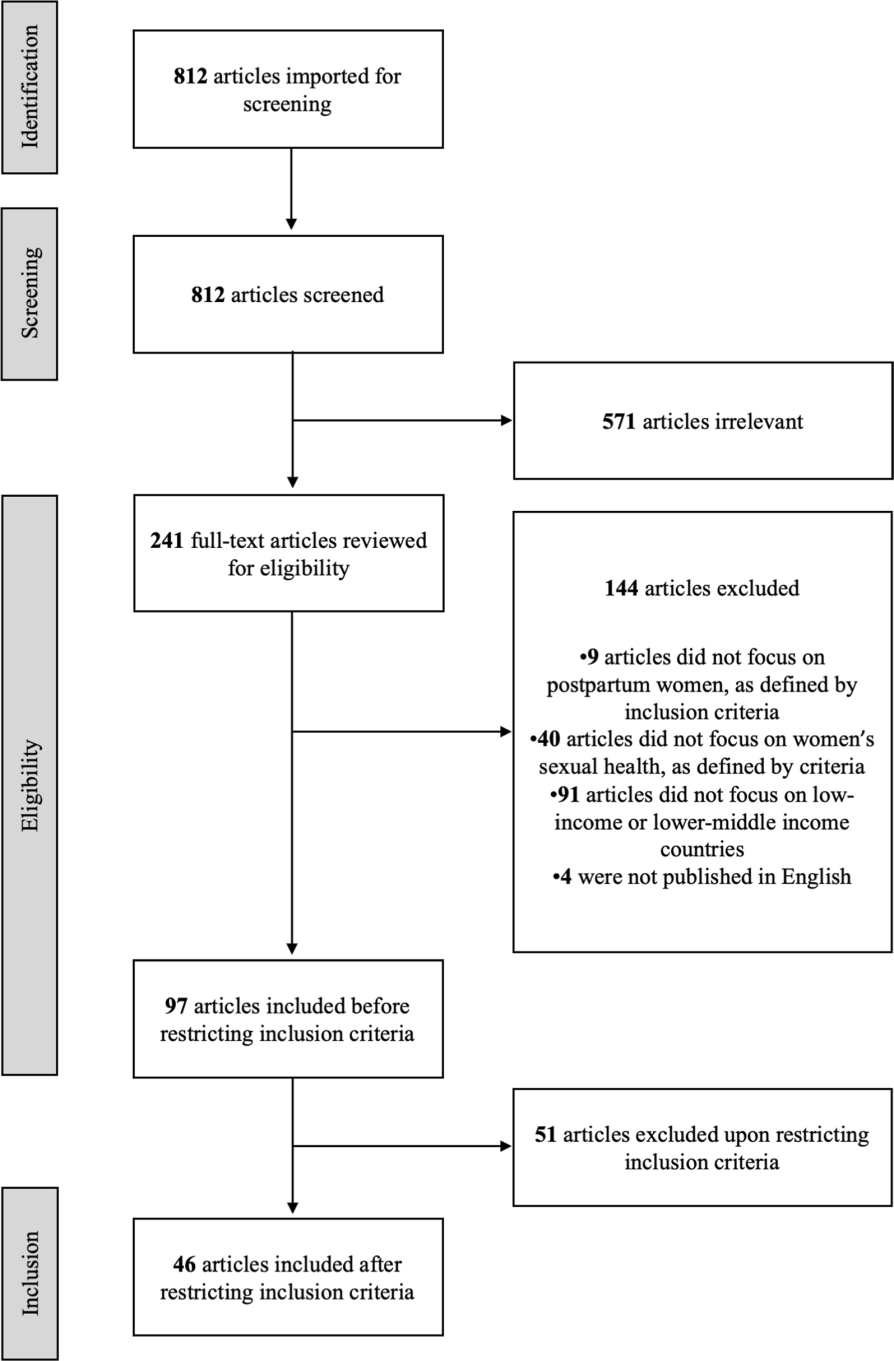
Flow diagram of scoping review process

The search yielded 812 results, all of which were screened via title and abstract review. Of these, 241 progressed to a full-text review. Based on the initially broad inclusion criteria, 97 articles were eligible for inclusion. After further restricting the inclusion criteria to only studies involving sexual function and/or dysfunction, only 46 articles were eligible and included (Fig. 1). Key characteristics of each article (author, year, objective, population, study design, sexual health topic, results) were described in Microsoft Excel tables by two Masters-level researchers and consolidated with input by the lead author; extracted data and study characteristics were then analyzed by positive sexual health (i.e., sexual function), negative sexual health (i.e., sexual dysfunction), and studies that reported both positive and negative sexual health or approached sexual health from a neutral perspective, which often examined resumption of sex as an outcome without indication of whether this outcome was viewed positively or negatively.

## Results

The final inclusion and exclusion criteria yielded 46 articles (Tables 1, 2, 3). Of these 46 articles, five studies focused on positive sexual health, 13 studies on negative sexual health, and the remaining 28 on both positive and negative sexual health or without specified directionality. The most common outcome examined was resumption of sex after childbirth. The majority of studies occurred within sub-Saharan Africa (n = 27), with additional geographic spread throughout the Middle East (n = 10), Asia (n = 5), and North Africa (n = 3); one study analyzed data from 17 Demographic and Health Surveys (DHS) [24]. Most studies were quantitative, including prospective cohort, cross-sectional, and clinical trial designs; however, eight studies solely or supplementally collected qualitative data.

**Table 1:** Studies on positive postpartum sexual health (n = 5)

Author (Year)	Location and study design	Objective(s)	Study population	Specific topic(s)	Key results
Anharan et al. (2015) [25]	Iran— Cross-sectional	To assess postpartum sexual function in mothers using different infant feeding methods	366 women referred to health centers in Mashhad; women who were receiving postpartum care, child growth monitoring, immunization, and family planning counseling 4 months after childbirth	Libido, Satisfaction, stimulation, Orgasm	– The mean total score of sexual function varied significantly between groups participating in different infant feeding methods (p = 0.04) – The exclusive breastfeeding group had the highest mean score for total sexual function, followed by the breastfeeding plus complementary feeding group, then by formula only, and lastly by breastfeeding plus formula – The four groups were also significantly different in terms of mean lubrication (p = 0.01) and satisfaction scores (p = 0.01); they were not significantly different in terms of desire, arousal, orgasm, or pain mean scores
Golmakani et al. (2015) [26]	Iran— Clinical Trial	To define the effects of an 8-week pelvic floor muscle exercise program on sexual self-efficacy in primiparous women after childbirth	79 primiparous women who were referred to health care centers in Mashhad, Iran in 2013, 8 weeks after delivery, to receive health care services	Pelvic floor muscle strength, Sexual self-efficacy	– Pelvic floor strength increased significantly among the intervention group only at 4 and 8 weeks after exercises (p < 0.001) – Pelvic floor muscle scores at 8 weeks were significantly different between the control and intervention groups (p < 0.001) – Sexual self-efficacy scores increased significantly for both the intervention (p < 0.001) and control (p = 0.001) groups at 4 and 8 weeks after the study. Sexual self-efficacy scores at 8 weeks were significantly different between the control and intervention groups (p = 0.001)
Mirzaei et al. (2021) [27]	Iran— Cross-sectional	To assess the impact of COVID-19 on psychological health, sexual function, and quality of life (QoL) in Iranian pregnant and lactating women and compare the results with non-pregnant/non-lactating women	604 pregnant and lactating women and non-pregnant/non-lactating women; May–June 2020	FSFI score items (desire, arousal, lubrication, orgasm, satisfaction, and pain)	– When comparing pregnant and lactating women, Female Sexual Function Index (FSFI) scores did not differ significantly for any sexual function domain. Total FSFI scores for pregnant and lactating women were nearly equivalent – When comparing lactating women with those neither lactating nor pregnant, FSFI scores for the following domains differed significantly: desire (p < 0.001), arousal (p < 0.001), orgasm (p = 0.007), and pain (p < 0.001) – The total FSFI score for lactating women was significantly lower than for those neither lactating nor pregnant (22.72 and 26.19 respectively; p < 0.001) – Among the lactating and pregnant women, 37% report sexual dysfunction related to lubrication, compared to 54% of women neither pregnant nor lactating (p < 0.001)
Nezhad & Goodarzi (2011) [28]	Iran— Cross-sectional	To gain insight regarding the perceptions of intimacy and sexuality held by postpartum couples, and the relationships of perceived sexuality, and intimacy levels on postpartum marital satisfaction	128 postpartum married couples of Ahvaz, Iran (6–36 weeks postpartum) obtained through cluster random sampling with the help of childbirth centers	Sexuality, Sexual satisfaction, Intimacy	– Most (91%) of the female participants reported that they felt healed by 8 weeks after delivery – Gender was significantly associated with sexuality, with the male participants reporting a higher level of sexuality than the female participants, but was not significantly associated with sexual satisfaction, intimacy, or marital satisfaction – Among participants, when total intimacy and sexual satisfaction are both high, marital satisfaction was also high for 100% of participants, vs. 68% when intimacy was high and sexual satisfaction was low (p = 0.006). Among participants with low total intimacy and high sexual satisfaction, marital satisfaction was still high – These correlations were identified in both couple and individual data and suggest that having a high level of intimacy may buffer the negative effect that low sexual satisfaction can have on marital satisfaction – Results also indicate thatwomen's satisfaction with their appearance is positively correlated with sexuality (p < 0.001) and that fatigue and sexuality are negatively correlated (p < 0.001)
Zamani et al. (2019) [29]	Iran—Clinical Trial	To investigate the effectiveness of sexual health counseling on woman's sexual satisfaction in the postpartum period	80 Iranian postnatal women (aged 18–35) who attended health-care centers 3 months to 1 year after childbirth in Mashhad, Iran in 2016	Sexual satisfaction	– At baseline, sexual satisfaction scores did not differ significantly between the intervention group and control group 8 weeks after the intervention; however, sexual satisfaction was significantly higher in the intervention group (p = 0.03) – Pre- and post-test sexual satisfaction scores differed significantly in the intervention group only (p < 0.001)

**Table 2:** Studies on negative postpartum sexual health (n = 13)

Author (Year)	Location and study design	Objective(s)	Study population	Specific topic(s)	Key results
Achour et al. (2019) [30]	Tunisia—Prospective Cohort	To investigate the psychosomatic impact of vaginismus in pregnant women and evaluate the quality of their therapeutic care in Tunisia	20 pregnant females with diagnosed vaginismus at the time they presented at an emergency department, followed into the postpartum period	Vaginismus	– Among participants who had a vaginal delivery, 75% considered their vaginismus to be cured following delivery; n = 4, however, experienced aggravated vaginismus symptoms following their vaginal delivery – Though recommended, no participants took part in pelvic floor training postpartum. Similarly, all participants were directed towards a sexologist for postpartum follow-up for their vaginismus, but none pursued: 20% were uninterested in resolving their vaginismus while 60% referenced that their sexual life was of minimal importance compared to motherhood – 70% reported feeling misunderstood by their health providers during pregnancy
Adanikin et al. (2015) [31]	Nigeria—Prospective Cohort	To determine the history of resumption of intercourse after childbirth and associated contraceptive practices among women in the southwest region of Nigeria	181 women with live births who delivered in an OBGYN teaching hospital in Ado-Ekiti—interviewed weekly until 6-months postpartum	Resumption of sex, Dyspareunia	– 27.6% of participants had resumed sexual intercourse within 6 weeks of childbirth, 63.3% within 3 months, and 70.2% within 6 months – The period prevalence of dyspareunia within 6 months of delivery was 36.2% – While 78.4% of participants who had had a vaginal delivery resumed sexual intercourse within 6 months of childbirth, significantly fewer (59.2%) of those who had had a caesarean section had resumed by this time – Resumption of sexual intercourse was not associated with perineal injury or experience of dyspareunia
Assarag et al. (2013) [32]	Morocco—Cross-sectional	To measure and identify the causes of postpartum morbidity 6 weeks after delivery and to compare women’s perception of their health during this period to their medical diagnoses	All women aged 18 to 49 in the Al Massira district who had delivered between December 2010 and March 2012 in the delivery house, hospital maternity wards, or private clinics (n = 1210)	Episiotomy, Prolapse, Infections	– 44% expressed one or more complaints at their postpartum consultation. Of those with a complaint, 91% did not consult with a physician about their complaint(s) – The most frequent complaint reported during a postpartum consultation was mental distress, followed by genital infections (including vaginal discharge and/or leaking), and breast problems – Additionally, 10% of participants reported other gynecological and obstetric complaints (including uterine prolapse, sexual problems, and infected episiotomy). Lastly, 2% of participants reported burning during urination and 1% reported urinary leakage – A higher prevalence of postpartum complaints was identified among women aged 30 and above, employed women, women that had delivered in the private sector or at home, and women with complications during delivery
Boene et al. (2020) [33]	Mozambique—Qualitative	To describe women’s experiences of antenatal, partum and post-partum care in southern Mozambique, and to pinpoint those experiences that are unique to women with fistula	14 women with a positive diagnosis of fistula and an equal number without, between the ages of 16 and 49	Obstetric fistula	– Among the 14 participants with a fistula diagnosis, six were reporting on their first birth, nine reportedly had a caesarean delivery, and 10 had a stillbirth – Most women with an obstetric fistula reported not having had sex since its onset – One woman reported that her husband had justified taking a second wife because of her fistula, which he viewed as a handicap
Ferdous et al. (2012) [34]	Bangladesh—Prospective Cohort	To investigate the association of postpartum maternal morbidities/disabilities with various acute obstetric complications arising during pregnancy or delivery, and with sociodemographics and other key characteristics of women at delivery	N = 1037 women with four categories of deliveries: uncomplicated normal vaginal birth, those who suffered a perinatal death, those who had severe or less severe complications during pregnancy or delivery, or those who had a C-section but no recorded maternal indication	Prolapse, Fistula, Infection, Perineal tear	– Sexual health outcomes varied significantly by the presence/type of delivery complication, including perineal tear (p < 0.001), clinically diagnosed genital infection (p = 0.04), and uterine prolapse (p < 0.001) – Participants who experienced delivery complications, compared to those who did not, were less likely to experience genital prolapse or perineal tearing – Participants with a perinatal death, compared to those with an uncomplicated birth, were more likely to be diagnosed with a genital infection and less likely to experience genital prolapse – Participants that had a cesarean section, compared to those with an uncomplicated birth, were less likely to experience genital prolapse – Perineal tearing was more prevalent among women over 30, among women with parity over four, among women in the poorest wealth quintile, among women who delivered at home, and among women that had a vaginal delivery – Prolapse was more likely to occur among women aged 20–29 and 30 + vs. women under 20, and among women with parity of 2–4 and 4 + vs. parity of 1. Prolapse was less likely among women who had a cesarean section vs. vaginal delivery – Perineal tearing was more likely to occur among: women aged 30 + vs. women aged < 20; women who delivered at home vs. in a hospital; and women with perinatal death vs. a live baby – Genital infection was more likely to occur among women with perinatal death
Gudu and Abdulahi (2017) [35]	Ethiopia— Prospective Cohort	To assess labor, delivery, and postpartum complications in nulliparous women with FGM/C and evaluate the attitude of mothers towards elimination of FGM	288 nulliparous women out of 1,125 mothers admitted for labor and delivery in the study period	FGM/FGC, Infection	– There was a 91.7% prevalence of female genital cutting (FGM/C). Of those who had experienced FGM/C, 7.6% had Type-II FGM/C, while 92.4% had Type-III. The age at which participants experienced FGM/C ranged from 2–9 years old – 90.3% of participants believed FGM/C to negatively impact labor and delivery. All participants who underwent FGM/C were fearful about problems that may arise during labor or delivery as a result of the FGM/C – Anterior episiotomy was needed to facilitate delivery for 83.0% of participants, all of whom had type-III FGM/C – In total, 29.0% of participants experienced spontaneous perineal tearing (31.1% with FGM/C; 8.3% without) – Postpartum complications occurred in 39% of participants (25.7% postpartum hemorrhage, 24% genital infection, and 12% psychological disturbance) – Postpartum hemorrhage was present in 27% of women with FGM/C and 8% of those without FGM/C – Postpartum infection was present in 14% of women with FGM/C and 4% of those without FGM/C
Islam et al. (2013) [36]	Pakistan—Randomized experiment	To assess the morbidity from episiotomy	100 patients who were given a mediolateral episiotomy (group I) and an equal number (group II) who delivered without episiotomy	Perineal tearing, Vaginal lacerations, Postnatal pain, Dyspareunia, Uterine prolapse	– Among patients with episiotomy, 69% reported postnatal pain, vs. 12% without episiotomy. Similarly, 69% of the episiotomy group reported dyspareunia, vs. 12% from without episiotomy group – No significant differences in pressure, incontinence, or uterine prolapse between those who received episiotomy and those who did not
Jambola et al. (2020) [37]	Ethiopia—Cross-sectional	To assess the early resumption of sexual intercourse (i.e., before 6 weeks postpartum) and associated factors among postpartum women attending public health institutions in Western Ethiopia	509 postpartum women who came for postnatal care or brought their babies for immunization to one of the participating public facilities 6 weeks after childbirth	Resumption of sex, Sexual Morbidities, Coercive sex/pressure to resume	– 20.2% of participants resumed intercourse during the first 6 weeks postpartum. Of those who resumed sex during the puerperium, 46.6% reported being pressured by their husband to resume intercourse – Few participants (16.9%) had received guidance or information about intercourse during the postpartum period – Among the sexually active participants, 22.4% reported one or more sexual morbidities or problems upon resuming intercourse. Problems included dyspareunia (41.7%), vaginal dryness (27.1%), reduced sexual desire (10.4%), vaginal bleeding (8.3%), abnormal vaginal discharge (6.3%), and vaginal tightness (6.5%) – Women who resumed sex early used contraception less frequently than those who resumed after 6 weeks postpartum (41.8% vs. 71.2%) – Among those who had not yet resumed sexual activity, reasons for abstinence included: feeling it was not yet acceptable to resume, the husband being unavailable, avoiding pregnancy, feeling unwell, religious reasons, being uninterested, and advice from a health worker – In multivariable analysis, the likelihood of having resumed sexual intercourse was significantly associated with: the mother having some secondary-level education (aOR 0.22), low parity (aOR 3.52), the husband having some elementary-level (aOR 0.23) or secondary-level (aOR 0.25) education, normal vaginal delivery (aOR 5.44), having a male infant (aOR 1.94), wanting another child (aOR 5.71), and being pressured by the husband to resume sex (aOR 9.89)
Lagro et al. (2003) [38]	Zambia—Cross-sectional	To know if women experienced health problems after childbirth, the specific problems they experienced, and if they did anything about them	Women who attended the hospital within three months after delivery of a live or stillborn baby with a gestational age of more than 22 weeks or weighing more than 500 g	Resumption of sex, genital tract infections, Breakdown of episiotomy/perineal tear, Various sexual health symptoms	– 27% of participants (between 6 weeks and 3 months postpartum) had resumed sexual intercourse; 90% had done so within 2 months after delivery – Vaginal discharge was reported by 31% of participants and abnormal vaginal bleeding by 7%; 21 participants seemed healthy, but upon physical examination revealed "pus-like" discharge or the breakdown of their perineal tear/episiotomy – Among participants for whom high vaginal swab results were available, 17% had abnormal results. The combination of the following symptoms (9% of all participants) was predictive of a puerperal infection: lower abdominal pain, badly smelling discharge, and fever – Among women who underwent a vaginal swab, physical examinations indicated vaginal discharge among 21% and tender uterus among 10%
Nazari et al. (2021) [39]	Iran—Cross-sectional and Qualitative	To determine the educational needs of mothers after childbirth	Quantitative: 250 pregnant mothers in the third trimester, in the first 48 hours after delivery, in the first 6 months after delivery, and in the second 6 months after delivery who were referred to five health centers in Bojnourd to receive midwifery care Qualitative pregnant women and postpartum women up to year after delivery, their spouses and key informants	General sexual health	– Qualitative themes highlight that sexual health needs during the postpartum period were often neglected. One participant, 37 years old and 2 months after delivery, discussed her experience of vaginal dryness, noting that she tried but "could not have sex" and utilized ineffective ointment – Incorrect beliefs and limited awareness were further obstacles to meeting sexual health needs. Some participants, for example, believed that having sex during pregnancy could negatively impact the fetus – The mean educational need scores for the area of sexual health were not statistically different between the four periods studied (pregnancy, 48 hours after delivery, the first 6 months postpartum, and the second 6 months postpartum; p = 0.12)
Oboro & Tabowei (2002) [40]	Nigeria—Panel study	This study addresses the postnatal sexual health of Nigerian women	122 married primiparas at the Kwale Zonal Hospitals, Delta State of Nigeria	Loss of sexual desire, Lack of vaginal lubrication, Lack of vaginal muscle tone, Vaginal tightness, Painful penetration, Painful intercourse, Difficulty achieving orgasm, Irritation or bleeding after sex, Coital frequency, Sexual satisfaction, Type of intercourse	– Reported sexual problems at 6 weeks, 3 months, 6 months postpartum, respectively: – Loss of sexual desire: 61%, 40%, 26% – Lack of vaginal lubrication: 51%, 29%, 13% – Lack of vaginal muscle tone: 22%, 17%, 10% – Vaginal tightness: 33%, 21%, 11% – Painful penetration: 47%, 30%, 21% – Painful intercourse: 55%, 34%, 19% – Difficulty achieving orgasm: 41%, 27%, 15% – Irritation or bleeding after sex: 19%, 9%, 6% – Following childbirth, 77% of women reported a decrease in coital frequency and 37% reported diminished sexual satisfaction. Following childbirth, the vaginal route became less frequently employed during intercourse (93% vs. 100% pre-childbirth, p = 0.004) – Following childbirth, sexual dysfunction overall generally increased (47% vs. 21% pre-childbirth, p < 0.001) – After completing the questionnaire at 6 weeks postpartum, 47 women initiated discussion of sexual matters – Dyspareunia at 3 months postpartum was significantly more likely among women who: perineal trauma (aOR 2.00) and pre-pregnancy dyspareunia (aOR 2.36) – While 68% or participants felt the need for assistance with postpartum sexual dysfunction, only 12% sought out help from healthcare professionals – Though postnatal clinic health professionals reportedly discussed contraception with 98% of the women, sexual health was only discussed with 29%
Surkan et al. (2017) [41]	Bangladesh—Prospective Cohort	To provide an initial estimate of the magnitude of depressive symptoms among women in the first year postpartum, identify risk factors and specifically to estimate strength of associations between several health conditions following childbirth and depressive symptoms	39,434 married women (ages 13–44) in 19 rural administrative unions in adjacent districts of Gaibandha and Rangpur in northwest Bangladesh who participated, during pregnancy and the early postpartum period, in the JiVitA-1 trial and gave birth to singletons	RTI, Uterine prolapse	– When adjusting only for sociodemographic variables, experiencing a reproductive tract infection (RTI) (RR = 1.29; p < 0.001) or uterine prolapse (RR = 1.46; p < 0.001) at three months postpartum increased risk for high depressive symptomatology at 6 months – When fully adjusting for all maternal illnesses, experiencing an RTI at three months postpartum does not significantly increase the risk for high depressive symptomatology at six months (RR = 0.90; p = 0.21), though experiencing uterine prolapse at three months still increased the risk for high depressive symptomatology at 6 months (RR = 1.20; p = 0.01)
White (2004) [42]	Cambodia–Qualitative	To detail the specific beliefs of Khmer women in Cambodia regarding postpartum, the taxonomies they use to describe postpartum conditions, and the practices they follow to prevent sickness and death	11 FGDs with 88 women of childbearing age and in-depth interviews with 21 women and 20 birth attendants. The childbearing age women included were ethnically Khmer, Khmer-speaking, had given birth within the last three years, and lived in the rice-growing basin of the Mekong and Tonle Sap Rivers	Resumption of sex, Sexual coercion/violence, Infection	– Resuming sex too soon was seen as the trigger for one type of toas (postpartum illness/morbidities) associated with symptoms of weakness, palpitations, abdominal cramps/pains, weight loss, and poor appetite – Toas damneyk more typically associated with the woman resuming sexual intercourse (often by force) before she felt ready, as opposed to merely resuming intercourse during "immature sawsaye" (the culturally understood period of postpartum recovery) – The primary symptom associated with toas damneyk was thinness. Other associated symptoms included dry skin, insomnia, a burning sensation in extremities, abdominal cramping, backbone stiffness, and "hotness in the body." Toas damneyk was treated in various ways, including burning the couple's pubic hair, adding it to rice wine, and drinking the concoction; consuming the water used to cleanse the male partner's penis; and other traditional medicines

**Table 3:** Studies on both positive and negative postpartum sexual health (or neutral; n = 28)

Author (Year)	Location and study design	Objective(s)	Study population	Specific topic(s)	Key results
Alum et al. (2015) [43]	Uganda—Cross-sectional	To assess prevalence and factors associated with early resumption of sexual intercourse among postnatal mothers	374 postpartum women who came for postnatal care or brought their babies for immunization (within 6 months postpartum) to one of three postnatal and immunization clinics of a teaching hospital	Resumption of sex	– 21.6% of participants had resumed sexual intercourse within 6 weeks of childbirth – Participants who were more likely to have resumed intercourse early included those that: had a high income, had low parity, had ever used contraception, or had a spouse with a high education level
Anzaku and Mikah (2014) [44]	Nigeria—Cross-sectional	To describe the current sexual practices of postpartum women, sexual morbidity, contraceptive prevalence and predictive factors for early postpartum sexual intercourse and associated sexual problems	340 women at a child welfare clinic 14 weeks after childbirth at a teaching hospital in Jos, Nigeria	Resumption of sex, Sexual morbidities	– Episiotomies were present in 19.7% of participants and vaginal lacerations in 29.9%. Most genital tract injuries (77.9%) had healed well; the rest led to complications including infection, chronic pain, and scarring – 67.6% of participants had resumed sex by 14 weeks after birth. The most prominent reason for resuming intercourse (reported by 77.4% of those who had resumed sex) was request of the husband; other reasons included: convenience (14.8%), advice given by health workers (4.3%), the woman's initiation (2.6%), and cultural demands (0.9%) – Reasons for not resuming intercourse included: husband's unavailability (reported by 38.2% of those who had not yet resumed), feeling that it was not yet time (21.8%), to prevent pregnancy (16.4%), being unwell (12.7%), uninterest (5.5%), cultural reasons (3.6%), health worker advised not to (1.8%) – Among those who had resumed coitus, 62.6% experienced one or more sexual morbidity upon commencing intercourse, though only 22.2% of these women were still experiencing the problem(s) when enrolled in the study. Only 5.6% of those with a sexual problem had pursued medical advice or treatment – Reported sexual problems included: vaginal dryness (16.7% of reported problems), deep dyspareunia (14.9%), vaginal tightness (13.1%), superficial dyspareunia (12.5%), loss of sexual desire (11.9%), vaginal looseness (8.3%), abnormal vaginal discharge (7.1%), vaginal bleeding (4.8%), tiredness (4.8%), and other problems (5.9%) – In multivariate regression, women that had a vaginal delivery were more likely to have reported one or more sexual problems when resuming sex (OR 3.6, p = 0.01); those who had a vaginal laceration or episiotomy were more likely to experience sexual problem(s) when resuming sex (OR 2.4, p = 0.04)
Asadi et al. (2021) [45]	Iran—Qualitative	To explore the experiences related to postpartum changes in women	23 women who have given birth and healthcare providers (midwives and obstetricians)	Perception of sexual dysfunction	– Postpartum participants described feeling less sexually attractive than in the past, referencing vaginal loosening as a reason, and felt that they were not well-groomed – Some postpartum participants described a decreased feeling of sexual attractiveness (and subsequent self-confidence) in relation to episiotomy scars and the protrusion of labia minora – Some postpartum participants reported that their husband's approval of these appearance changes resulted in greater sexual performance and less anxiety – Postpartum participants also described not enjoying sex because of dyspareunia, as well as due to feelings of burning/dryness, and reported feeling less sexual desire during the postpartum period
Borda et al. (2010) [24]	17 DHS countries—Cross-sectional	To identify factors affecting return to sexual activity and use of modern family planning among women in the extended postpartum period	15 of 17 countries included all women, two included married women only	Resumption of sex	– In all 17 countries, women who were 0–2.9 months postpartum reported the least sexual activity – In 13 countries, the majority of women 3.0–5.9 months postpartum had resumed sexual activity and over three-quarters of women 9.0–11.9 months postpartum were sexually active – In 10 countries, exclusive breastfeeding was significantly associated with the woman's resumption of sexual activity, with women currently breastfeeding less likely to have resumed sexual activity at the time of the survey – In 16 countries, resumption of sexual activity was significantly associated with the return of menses. In 14 of these countries, the odds of having resumed sexual activity among women whose menses had returned was more than double the odds of resumption among those whose menses had not returned – Having resumed sexual activity was associated with the duration of the postpartum period (with 0–2.9-month period as referent); in all but Zambia, this association was significant for two or more of the postpartum intervals
Dadabhai et al. (2020) [46]	Malawi—Prospective Cohort	To determine time from delivery to resumption of sexual activity and menses by HIV infection status	878 women (460 HIV-uninfected and 418 HIV-infected) who attended at least one follow-up visit	Resumption of sex	– Comparable proportions of HIV-infected and HIV-uninfected women reported sexual activity at each visit (< 6.0% at the 6-week postpartum, increasing to > 82.0% at the 12-month) – Marital status was the only variable significantly associated with early resumption of sex and this significance remained for both groups after stratifying by HIV infection status
Desgrées-du-Loû and Brou (2005) [47]	Cote d'Ivoire—Qualitative	To understand how couples negotiate the resumption of sexual relations following childbirth, the role of post-partum sexual abstinence, the underlying norms, who makes the decision and what each partner's agency is in the decision	Men (n = 10) and women (n = 23); parent of an already weaned child under the age of five	Resumption of sex, Coercive sex, Fear of STIs/HIV	– One argument for sexual abstinence was breastfeeding-related taboos: most women referenced breastfeeding (specifically the belief that semen should not mix with breastmilk, for the health of the child) as a reason for sexual abstinence; nine women and eight men reported resuming sex only after the complete cessation of breastfeeding – Another common practice was waiting to resume sex until the child could walk; this practice overlapped with breastfeeding-related taboos, given the idea that the ability to walk suggested resilience and no more need for breastfeeding – For some, resumption of sex depended on the recuperation of the mother from childbirth; the recuperation period ranged from 5–8 months or until menses returned. This practice was based both on desires to regulate fertility and concern for the mother's health – Women that spoke to a friend of sister about postpartum abstinence were warned about the risk of the husband's unfaithfulness (if abstinence lasted too long) and the risk of pregnancy (if abstinence was too brief) – All of the women reported that the resumption of sex was generally initiated by the man; eight women reported feeling pressured to resume sex earlier than they wanted to – Some women felt that polygynous marriages allowed for longer abstinence, given sexual activity with other wives – Many women expressed fears of infidelity by their partner and related STI and/or HIV risk – Women seemed to have some agency regarding the resumption of sex: many postponed resumption a few months following their husband's request; some only agreed to resume sex with protection (condoms were believed to prevent semen mixing with breastmilk); some women moved in with their parents for the first year postpartum and/or avoided spending the night at their partner's house until ready to resume sex
Ezebialu and Eke (2012) [48]	Nigeria—Cross-sectional	To determine the average time for resuming vaginal intercourse during the puerperium, as well as factors that are associated with resumption of coital activity	860 mothers in a postnatal clinic at their first visit postpartum	Resumption of sex, Fear of pain/experienced pain	– By the time of their first postnatal visit, 29.7% of participants had resumed vaginal intercourse. Factors that varied significantly between those who had and had not resumed coitus by their first postnatal visit included: resumption of menses (p < 0.001), place of residence (p = 0.001), HIV status (p = 0.004), having minimal or no education (p = 0.009), mode of delivery (p = 0.039), extremes of age (p = 0.007), and being married (p = 0.002) – In multivariate analysis, HIV-negative status and the resumption of menses showed strongest associations with resuming sex during the puerperium (p < 0.001) – Among those who had not yet resumed sex, reported reasons for abstaining included avoiding pregnancy (64.5%), partner unavailable (14.9%), lack of interest (9.1%), fear of pain at the site of episiotomy (2.5%), and no reason (11.6%) – The most common reasons justifying recommended durations of postpartum abstinence were to allow for the woman's full recovery (39.5%) and for family planning purposes (27.9%) – Six women reported experiencing pain during sex
Gadisa et al. (2021) [49]	Ethiopia—Cross-sectional	To assess the early resumption of postpartum sexual intercourse and its associated risk factors among married women who visited public hospitals for child immunization services	330 postpartum women, 14 weeks after childbirth, randomly selected	Resumption of sex	– 53.9% resumed sex early (< 6 weeks postpartum); 14% of these resumed sex < 4 weeks postpartum – 77% reportedly resumed sex at their husband's request – Factors significantly associated with early resumption of sex included: low income (aOR 0.19), monogamous marriage (aOR 3.78), having practiced sexual intercourse during pregnancy (aOR 4.55), having had a cesarean delivery (aOR 0.06), and using contraception (aOR 3.7)
Glynn et al. (2001)[50]	Multi-country (Cameroon, Zambia, Kenya)—Cross-sectional	To demonstrate if prolonged postpartum sexual abstinence may increase the risk of HIV through an associated increases in male extramarital sexual contacts	From six antenatal clinics: n = 1532 women in Yaoundé, Cameroon; n = 1480 in Kisumu, Kenya; n = 1021 in Ndola, Zambia	Postpartum abstinence	– Postpartum abstinence > 6 months was reported by half of participants in Yaoundé, 17% in Kisumu, and 13% in Ndola; abstinence > 12 months was reported by 23% in Yaoundé, 8% in Kisumu, and 4% in Ndola – Postpartum abstinence was shorter among married women and similar between polygamous/monogamous across sites – In Yaoundé, postpartum abstinence duration was strongly associated with HIV seropositivity overall, though when restricting to those who were married to their current partner at time of birth results remained only borderline significant – In Yaoundé, postpartum abstinence was longer among women with prolonged postpartum amenorrhea – In Kisumu, postpartum abstinence was not associated with HIV status. In Ndola, postpartum abstinence duration was slightly positively associated with being HIV-positive overall, though not when restricting to married women
Iliyasu et al. (2006) [51]	Nigeria—Cross-sectional	To assess contemporary postpartum beliefs, practices, and health problems of mothers in a typical Hausa rural community	300 mothers with children under five	Resumption of Sex, Perineal pain, Harmful traditional practices	– 76.7% of participants resumed sex 6–11 months after delivery – Certain postpartum rituals and cultural practices were believed to strengthen the mother and return her stamina (believed by 92.7%), help heal perineal wounds (77.3%), stimulate lactation (81.3%), and aid in the drainage of lochia (66.0%). According to 64% of participants, non-observance of such practices could result in body swelling and, according to 4%, to perineal pain and foul-smelling lochia – Few participants (8.7%) did not believe that the practices were beneficial. Significantly more women with a formal education (22.5%) did not believe the practices to be beneficial compared to those without any formal education (2.8%; p < 0.01) – Such commonly practiced rituals included: confinement for 40 days after birth (practiced by 87.7%), confinement beyond 40 days (12.3%), hot ritual baths (86.0%), nursing in heated rooms (84.3%), laying on heated beds (5.3%), eating a gruel dish enriched with salt (82.7%), and eating spicy foods (85.3%) – Some common postpartum complaints included excessive bleeding (reported by 16.3%) and lower abdominal/perineal pain (56.3%); most women who reported a complaint (66.0%) sought traditional remedies, while 25.0% pursued modern medical care and 9.0% did not utilize any medication – Nearly half of participants stated that they would continue practices regardless of their harmful effects, 25% felt that the practices should be optional, and 8% supported discontinuation
Iliyasu et al. (2018) [52]	Nigeria—Cross-sectional	To determine the prevalence of postpartum sexual activity, delivery-coitus interval, and their determinants among women who delivered within 12 months of the study and attended the postnatal/family planning and child welfare/immunization clinics at Aminu Kano Teaching Hospital in Kano, Nigeria	317 mothers attending the postnatal/family planning clinics and those who brought their children for immunization	Resumption of sex, Vaginal discharge, Vaginal irritation, Lack of libido, Dyspareunia	– 52.1% of participants underwent episiotomies. Genital injuries were present among 15.1%, most commonly vaginal lacerations (70.8%) and perineal tears (25.0%) – 66.9% had resumed sex by study. The duration of postpartum abstinence ranged from 4–28 weeks (mean = 9.6) – Among sexually active, primary reasons for resuming sex were husband's demands (67.5%) and own desire (14.6%) – Among those still abstaining from sex, reasons for abstinence included: the infant being too young (81.0%), avoiding pregnancy (13.3%), lack of interest (2.9%), and being either divorced or widowed (2.9%) – Most sexually active participants reported experiencing a sexual problem (64.2%) including: dyspareunia (32.5%), diminished sexual desire (31.6%), vaginal dryness/soreness (15.6%), vaginal 'looseness' (15.6%), and discharge (6.1%) – Fewer than two-thirds of those experiencing a sexual problem sought help; most commonly sought sources of advice were friends, mothers, physicians, and internet – Reasons referenced for not seeking guidance included feeling shy, problem resolving on its own, cultural/religious factors, and not having a female doctor to ask – Upon adjusting for confounders, the following characteristics remained significantly associated with having resumed sex: not co-habitating with husband (aOR 0.47, p = 0.001), spontaneous vaginal delivery (aOR 1.10, p = 0.05), infant's age 1–6 months (aOR 1.53, p = 0.02) or 6 + months (aOR 2.10, p = 0.001), having five or more living children (aOR 1.21, p = 0.03), and not having resumed menstruation (aOR 0.34, p = 0.003)
Kinuthia et al. (2017) [53]	Kenya—Prospective Cohort	To characterize frequency and types of sexual behaviors and vaginal practices among HIV-uninfected women during pregnancy and up to 9 months postpartum	1252 pregnant, HIV-uninfected women who attended antenatal care clinics	Resumption of sex, Forced sex, Vaginal washing/ drying	– From 2 to 36 weeks postpartum, the proportion of sexually active women increased from 8% to 701% (p < 0.001) and the proportion of women who reported condomless sex within the past month rose from 6% to 60% (p < 0.001) – 60.1% reported vaginal washing, and prevalence was stable throughout follow-up. Vaginal washing at 36 weeks postpartum was associated decreased odds of having a partner of unknown HIV status, being a housewife, and longer relationship duration, and increased odds of history of abnormal discharge, later sexual debut, and past-month forced sex – Proportion of vaginal drying decreased over follow-up, and increased with increasing age **– **At 36 weeks postpartum, the odds of condomless sex (vs. protected sex or no sexual activity) in the past month was significantly higher among women who: were older, married, had more lifetime sexual partners, reported past-month forced sex, reported past-month anal sex, were unemployed, had HIV-uninfected partners, and had had more live births – Earlier resumption of sex after childbirth was associated with: older age, more lifetime sexual partners, partner's age compared to the woman's age, a history of anal sex, being marriage, whereas decreased hazard ratios were observed for having completed primary education or beyond, having had a caesarean section. having a partner with unknown HIV status, and partner circumcision status
Lundberg and Trieu (2011) [54]	Vietnam—Cross-sectional and Qualitative	To describe cultural beliefs and practices related to the postpartum period among Vietnamese women in Ho Chi Minh City	115 Vietnamese women, 95 in the first group and 20 in the second group. A questionnaire was used with the first group and a semi-structured in-depth interview was used with the second group	Resumption of sex, Fear of prolapse, Perineal wounds	– All participants abstained from sexual intercourse with their spouses for the first 3–4 months following childbirth – Participants believed that engaging in sex too early could negatively impact their health and/or perineal wounds – Participants also feared the possibility of earlier sex leading to uterine prolapse or pregnancy – Some participants were not interested in having sex with their spouses because they were very tired from caring for their infants
Maamri et al. (2019) [55]	Tunisia—Cross-sectional	To evaluate the sexual function of a population of women in postpartum, and identify possible particularities and associated factors of the studied population	100 women who gave birth 6 months prior	Resumption of sex, FSFI score items (desire, arousal, lubrication, orgasm, satisfaction and pain), Hypoactive sexual desire, Postpartum sexual practices, Perineal trauma, & Other sexual morbidities	– Among vaginal births, 42/53 had perineal lesions – The mean duration of postpartum abstinence was 2.1 months – Among those who underwent a scheduled cesarean section, 45% resumed sex 4–6 weeks after delivery, while 72% of those who had an instrumental vaginal delivery resumed at six months postpartum or later (p = 0.01) – Reasons for engaging in sexual activity included both partners' pleasure (83%), only the husband's pleasure (13%), and personal pleasure exclusively (5%). Additionally, 10% of participants report having sex due to marital duty and 6% to avoid infidelity by the husband – Reasons offered for abstaining from sex included fear of pain (32%) and fear of repeat pregnancy (13%). Feeling physically undesirable (37%) and feeling their body had changed (31%) led women to feeling disconnected from their bodies. Other hinderances included excessive fatigue (24%), the presence of a child (50%), and mother's unavailability (60%) – 79% reported that their relationship changed postpartum – Hypoactive sexual desire was reported by 31% of participants – Most women (65%) reported achieving adequate lubrication always, almost always, or usually – The frequency of reaching orgasm was unaltered after delivery among 58% of participants, reduced for 29%, and rose for 13% – Sexual satisfaction did not change postpartum for 52% of participants, reduced for 33%, and improved for 15% – 14% of women reported dyspareunia
Mbekenga et al. (2011) [56]	Tanzania—Qualitative	To explore and describe postpartum experiences of first-time mothers in a Tanzanian, multiethnic, low-income suburb	10 first-time mothers recruited at two RCH clinics in the lower-income areas of Ilala municipality, Dar es Salaam city, Tanzania. They were recruited when bringing their infants for routine examination and vaccination 4–10 weeks after childbirth	Resumption of sex, Episiotomy	– Women expressed uncertainty and a need for information regarding care for episiotomy wounds – The women reported that reproductive and child health clinics did not provide enough health education, while antenatal clinics provided too much information all at once – Except for one, all the mothers planned to abstain from intercourse until ceasing breastfeeding (up to 4 years). A common belief is that having sex while still breastfeeding would make the infant ill and hamper their development – The mothers, however, doubted that their partners’ would be able to abstain from sex for this long; they believed that their partners may have sex with other people and risk contracting HIV. One participant, per an interview excerpt, described feeling strongly that her and her partner need to get HIV tested due to possible infidelity by her partner
Mekonnen (2020) [57]	Ethiopia—Cross-sectional	To assess the early resumption of sexual intercourse after childbirth and associated factors among women in the extended postpartum period in Gondar city, Northwest Ethiopia	634 women of child-bearing age who gave birth within the 12 months preceding the study period in Gondar city, Northwest Ethiopia	Resumption of Sex, Sexual coercion, Painful sex, Sexual desire, Vaginal dryness	– 61.4% of participants had vaginal delivery without an episiotomy/tears, 11.8% had a vaginal delivery with episiotomy/tears, and 6.5% had an instrumental delivery (forceps/vacuum); the remainder had a cesarean section – At the time of study, 89.7% had resumed sexual intercourse; of these, 26.9% had resumed sex before 6 weeks postpartum – The most frequent reason for resumption of sex was partner demand (reported by 76.1%) – Upon resuming sexual intercourse, 69.2% reported having no problems, 15.6% reported pain during sex, 6.0% reported vaginal bleeding/discharge, 5.1% reported a lack of desire, and 4.0% reported vaginal dryness – 29.7% of those who did experience a problem after resuming sex sought medical advice for their sexual problem – 28.5% had ever been advised about sexual activity – In multivariable logistic regression, the following were significantly associated with early resumption of sex: urban residence (aOR 6.12), low parity (aOR 2.26), partner demand as the reason for resuming sex (aOR 2.66), using any family planning (aOR 2.72), and obtaining postnatal care (aOR 1.45)
Nkwabong et al. (2019) [58]	Cameroon—Retrospective cohort	The study enquires whether nursing mothers regularly resume sexual intercourse before the 42nd day postpartum	120 women at their 6-week postpartum check-ups who delivered between 11/15/2013 & 12/31/2013	Resumption of sex, Perineal tear/pain, STI risk	– 79.1% of the participants had resumed sexual intercourse within the first 6 weeks after childbirth – Among those who resumed intercourse, 3.2% delivered via cesarean section vs. 56% of those that had not yet resumed sex (p < 0.001). Similarly, vaginal and uncomplicated deliveries both increased the likelihood of having resumed sex before the 6-week postpartum visit (p < 0.001) – Among those who had resumed intercourse, perineal trauma was present for 12.6% (including four episiotomies and eight perineal tears), vs. 60% of those who had not yet resumed sex (including two episiotomies and 13 tears) (p < 0.001)
Nolens et al. (2018) [59]	Uganda—Prospective cohort	The aim of this study was to assess how vacuum extraction was experienced by women after its re-introduction in a tertiary referral hospital in sub-Saharan Africa, using women-centered outcomes such as birthing experience satisfaction; pain 1 day after birth; and quality of life, pain and dyspareunia 6 weeks and 6months after birth	Women who gave birth (to a singleton in cephalic presentation) via vacuum extraction or SSCS at the main labor ward of the Mulago National Referral Hospital in Kampala, Uganda. Participants were interviewed at 1 day, 6 weeks, and 6 months after birth	Resumption of sex, Dyspareunia	– At baseline, 90.7% of participants had a known perineal status; of these, 35.9% had an episiotomy, 34.0% had an intact perineum, 29.8% had a first or second degree tear, and 0.8% had a third degree tear – Women who underwent vacuum extraction, vs. second– stage caesarean section (SSCS), experienced less pain in the first 24 hours following birth (p < 0.001), less vaginal/abdominal pain at 6 weeks postpartum (p < 0.001), and a similar amount of vaginal/abdominal pain at 6 months postpartum (p = 0.05) – At 6– weeks postpartum, no pain was reported by 76.2% of women who had a vacuum extraction, vs. 55.5% of those that had a SSCS (OR 2.56). Among women that had undergone vacuum extraction, pain scores indicated 'severe' or 'very severe' pain in the last 4 weeks for 3.4%, vs. 17.1% for those that underwent a SSCS (OR 0.17). For 50.0% (vacuum extraction), vs. 73.6% (SSCS), pain had interfered with their daily activities during the preceding 4 weeks (OR 0.36) -At 6-weeks postpartum, women who underwent vacuum extraction, vs. SCSS, had higher odds of having resumed sex (40.0% vs. 28.3%, OR 1.69, p = 0.01)
Odar et al. (2003) [60]	Uganda—Cross-sectional	To establish the time taken to resume sexual intercourse, the sexual morbidity associated with resumption, and the prevalence of sexual problems encountered by postpartum women attending immunization clinics in Mulago Hospital in Uganda	216 women, 3–6 months postpartum, who had brought their infants to the Mugalo Hospital immunization clinics for vaccination	Resumption of sex, Dyspareunia, Other sexual morbidities	– Among participants, 39.4% had either had an episiotomy or vaginal laceration; 87.2% of the episiotomies healed well while 12.8% became infected – At the time of the study, 66.4% of participants had resumed having sexual intercourse; 49.3% of these resumed sex during the puerperium. Of those who resumed during the puerperium, 8.5% resumed within the first week after birth. Time to resumption ranged from the first week to the 24th week after birth, with an mean time of 7.87 (± 4.9) weeks – Advice provided to mothers by health workers at discharge was inconsistent; only some advised to wait 6 or more weeks – Reasons for resuming sexual activity included: husbands' demands (46%), advise from health workers (7%), convenience (37%), and fulfilling cultural demands (10%) – Some cultural demands included an expectation for women to resume sexual activity within a week of delivery as to help heal her wounds and bring health to the infant. Some women resumed sex early because "they were entering a new house" – Reasons for not resuming sex included: not well yet (18%), health advice (37%), not interested (22%), and "husband's way" (23%). Some participants (n = 26) had not resumed sex because they had not been told when it would be safe to do so – 22.2% of participants experienced significant degrees of morbidity within 6 months of giving birth, including: dyspareunia (62.5%), vaginal discharge (18.8%), vaginal bleeding (15.6%), and tears or bruises (3.1%) – Early resumption of sex (within 6 weeks) was significantly associated with: having up to a primary-level education (OR 1.53; p = 0.03), having had a vaginal delivery (OR 3.84; p = 0.0005), and having an intact perineum (OR 1.73; p = 0.02) – Only 19 of the 32 women with morbidities sought help. The women who sought help were generally older (p = 0.01) – Women who sought help still felt shy to discuss their problems. Most sought advice and/or treatment from less qualified personnel, while the rest administered treatment themselves or reported that the problem ended within 1 day
Osinde et al. (2012) [61]	Uganda—Cross-sectional	To assess the factors associated with resumption of sexual intercourse and use of contraception in the 6-week postpartum period among women attending postnatal care at Kabale Regional Hospital, Kabale, Uganda	131 women who attended the hospital's postnatal clinic	Resumption of sex; Sexual coercion; Perineal wounds	– Among the 131 participants, 58.0% resumed sexual intercourse early (within 6 weeks following childbirth) – Education level of the spouse was significantly associated with early resumption of intercourse (aOR 0.2; p = 0.05) – Participants reported spousal pressure and the fear of their spouse leaving them as reasons for resuming intercourse early – Those who waited at least 8 weeks before resuming intercourse reported the following reasons for waiting: unhealed perineal wounds, the fear of conception, and geographic separation from their spouse
Rezaei et al. (2017) [62]	Iran—Cross-sectional	To evaluate women’s sexual function in the postpartum period in Iran	380 postpartum women attending 10 urban health centers	FSFI score; Sexual dysfunction was classified by FSFI score of ≤ 28	– 76.3% of participants reported sexual dysfunction – 79% of those with sexual dysfunction reported a lack of desire – In multivariate regression, primiparous mothers (aOR 1.96, p = 0.006) and those that exclusively breastfed their infants (aOR 2.47, p = 0.01) were more likely to report sexual dysfunction
Shabangu and Madiba (2019) [63]	Eswatini—Qualitative	To explore the practice of post-partum sexual abstinence in Swazi women and examine how cultural beliefs influence and promote the perpetuation of the practice	15 postpartum women selected via purposive sampling at health facility	Resumption of sex (sexual coercion and HIV transmission risk also mentioned)	– At interview, most women had yet to resume sexual intercourse; most of those that had resumed did so when their infant's age reached 6 months – Participants overall felt that postpartum sexual abstinence benefited the mother, infant, and spouse. They indicated that abstinence supports the mother's recovery from birth and allows her to adequately care for the infant – Participants believed that resuming sex early could hinder the infant's health, development, and growth as well as contaminate breastmilk, making it unhealthy for the infant to consume – Participants also reported being told by older women that early resumption of intercourse could result in their partner's illness and possibly even death – The baby's gender also influences the duration of abstinence; for boys, the period of abstinence is more relaxed (shorter) due to the higher social value placed on boys – Participants reported feeling pressure to abstain from sex from family members and in-laws; while in-laws typically enforced abstinence upon married women, abstinence was enforced by the woman's mother among single women – Women also expressed a lack of control over abstinence; they did not live with their partners during the abstinence period and felt that if they had, then they would have resumed sex early – Disadvantages of the culturally prescribed period of postpartum abstinence include potential risk of HIV transmission (given the lack of pressure placed on the men to abstain from pursuing sexual relations with other women)
Sheikhi et al. (2020) [64]	Iran—Randomized controlled trial	To determine the effect of sexual health education on sexual function and the time of sexual intercourse resumption in primiparous women referring to the health-care center of Zahedan, Iran	Primiparous women referred to health centers for postnatal care 3–5 days after birth, with healthy perineum or low-grade tears. Randomly assigned to the intervention or control group (n = 47 per group) with posttest at 8 weeks postpartum	Resumption of sex, Perineal status, Sexual desire/arousal, Vaginal lubrication, Orgasm, Sexual satisfaction, Dyspareunia	– 92/94 participants had had an episiotomy; two had grade one or two perineal tears – In the intervention group, the mean total sexual function (SFI) score increased from 12.7 (pre-intervention) to 17.4 (post-intervention) (p < 0.001); control score decreased slightly – Intervention and control group post-test scores were significantly different (p < 0.05) for all dimensions of sexual function, including: desire (p = 0.001), arousal (p = 0.009), lubrication (p = 0.001), orgasm (p = 0.001), satisfaction (p = 0.001), and dyspareunia (p = 0.003)
Shirvani et al. (2010) [65]	Iran—Cross-sectional	To investigate the sexual function of mothers at 1 year postpartum and associated factors	490 Iranian women who were recruited randomly at four time periods from childbirth: first 3 months, 4 to 6, 7 to 9 and 10 to 12 months, from January to July 2008	FSFI score items (desire, arousal, lubrication, orgasm, satisfaction and pain)	– The mean duration of postpartum abstinence was 57.17 (± 27.95) days from delivery – 52.9% abstained from intercourse beyond 45 days postpartum, possibly reflecting the common recommendation in Iran that intercourse be delayed until 6-weeks postpartum – Reasons for abstaining or delaying sex included: fear of pain (8.6%), having no interest (3.5%), avoiding pregnancy (2.5%), tiredness (2.2%), and bleeding (0.6%) – While 8.8% of participants reported sexual problem(s) and 40% noticed a reduction in sexual desire from pre-pregnancy to postpartum, only 2.4% of participants spoke to a health professional about their sexual problem(s) – Mean scores for all domains of sexual function and total sexual function varied significantly across the four postpartum intervals overall – The following sociodemographic characteristics were significantly correlated with sexual function: postpartum duration (r = 0.17; p = 0.0001), higher maternal age (r = − 0.12; p = 0.006), number of children (r = − 0.09; p = 0.02) and marriage duration (r = − 0.08; p = 0.05)
Sule-Odu et al. (2008) [66]	Nigeria—Prospective cohort	To review the postpartum sexual practices in the community, predominantly inhabited by the Yorubas	371 mothers who had just given birth to singleton babies and previously breastfed at least one child >= 6 months	Resumption of sex	– During the first month following childbirth, 84.6% of participants abstained from sexual intercourse; dropped to 2.1% by the 11th–15th months – Examined patterns by social class, age, and breastfeeding
Ugwu et al. (2021) [67]	Nigeria—Prospective cohort	To determine the comparative effect of caesarean section and vaginal delivery on female sexual function disorders	Postnatal mothers at two hospitals in Nigeria The intervention group of women delivered via caesarean section whereas women in the control group had a vaginal delivery (50 in each group)	Resumption of sex, Sexual Function Disorders	– Among the 48 participants that had a vaginal delivery, the perineum was intact after childbirth for 11, while 19 experienced perineal tearing and/or an episiotomy – Among those who had a cesarean section, 74.4% resumed intercourse by 6-weeks postpartum, vs. 52.1% of those who had a vaginal delivery (RR = 1.43; p = 0.03) – Those who had a cesarean section had significantly higher mean scores (p < 0.05) for the following sexual function domains: orgasm, pain, and satisfaction
Zulu (2001) [68]	Malawi—Mixed-methods	To examine ethnic differences in the tradition of postpartum sexual abstinence by comparing its observance and rationale in three culturally different ethnic communities in Malawi, using quantitative and qualitative data collected in those communities	Women's data from the three rural census enumeration areas (n = 273 south; n = 550 central; n = 288 north). In-depth interviews (IDIs) and focus group discussions (FGDs) conducted with key informants (n = 22 FGDs, n = 61 IDIs)	Resumption of sex	– Quantitative data suggest that postpartum sexual abstinence practices did not change substantially between 1988 and 1998 – Based on 1998 data, at 2 and 4 months postpartum, abstinence is similar in the north and in the south, much lower in the central region. Observed regional variation in postpartum abstinence duration corresponded with regional differences in knowledge of and adherence to customs requiring abstinence while breastfeeding and until after the resumption of menses – Participants elucidated beliefs related to the infant's nutrition and health in relation to postpartum abstinence. Participants believed that intercourse (via the exposure to semen) can contaminate breastmilk – Participants from each region studied also expressed that postpartum abstinence protected the male partner's health. It was perceived that postpartum bleeding ends when the mother's reproductive system has "cooled," signifying that she is free from harmful fluids. Participants listed a variety of symptoms men may experience when exposed to reproductive blood, some participants suggesting that the symptoms can resemble those of AIDS and others explaining that the effect can be fatal. These perceived symptoms also included sterility and the gradual loss of sexual prowess. Among participants in the central and southern regions, concerns were also mentioned in relation to abstinence after the postpartum bleeding period – The recommended duration of postpartum abstinence varied. Most participants from the central region recommended 3 and 6 months, though some indicated that one should abstain until the infant reached a certain level of physical maturity – Participants in the central and southern regions described various versions of child-strengthening rituals, which were performed before resuming intercourse as to protect the child from harm; participants explained that men too were advised to abstain from intercourse (including with other women) prior to a child-strengthening ritual – In the north there was a clear and definitive event (i.e. the resumption of menses) believed to indicate that resuming sex was then safe; northern participants believed that menstruation signifies that the woman's body is recovered and physically prepared for another pregnancy. Child-strengthening rituals were also performed in the north prior to resuming intercourse, as to protect the child's health. Participants explained that becoming pregnant before resuming menses and performing the ritual was considered disgraceful and appalling; parents who resume sex before this point were seen as callous and selfish. Older women sometimes judged parents' adherence to abstinence expectations by assessing the child's health

### Positive sexual health

Five studies examined positive sexual health during the postpartum period with outcomes including libido, satisfaction, stimulation, orgasm, pelvic floor muscle strength, sexual self-efficacy, female sexual function (measured via the Female Sexual Function Index (FSFI)), and intimacy [25–29] (Table 1). Notably, all five studies were conducted within the Islamic Republic of Iran and largely within the context of interventions or utilizing comparison groups. Specifically, Golmakani et al. examined the impact of a pelvic floor muscle exercise program on pelvic floor strength and sexual self-efficacy and found significant increases in both outcomes within the intervention compared to the control group [26]. Zamani et al. examined the effectiveness of sexual health counseling and found increased sexual satisfaction among intervention participants [29]. Two additional studies examined sexual function in relation to infant feeding practices, with mixed results [25, 27]. Additionally, Nezhad and Goodarzi examined intimacy and sexuality within the context of partnerships and found that having a high level of intimacy could potentially buffer against negative effects of low sexual satisfaction on overall marital satisfaction [28].

### Negative sexual health

Thirteen studies explored negative sexual health outcomes, including vaginismus, dyspareunia, episiotomy, perineal tears, prolapse, infection, obstetric fistula, female genital cutting, postnatal pain, uterine prolapse, coercion to resume sex, sexual violence, and loss of sexual desire/arousal [30–42] (Table 2).

Dyspareunia, or painful intercourse, was one of the most examined negative sexual health outcomes for postpartum women. In Nigeria, Adanikin found that over one in three women reported dyspareunia within six months after delivery [31]. Similarly, in Ethiopia, approximately one in five women reported sexual morbidities upon resuming intercourse in the postpartum period, and dyspareunia was the most common morbidity reported [37]. In Pakistan, dyspareunia was examined in relation to episiotomy, where dyspareunia was more prevalent among episiotomy patients than those without (69% vs. 12%) [36]. Further, in Nigeria, Oboro and Tabowei found that painful intercourse decreased throughout the postpartum period, with approximately 55% of women reporting painful intercourse at 6-weeks postpartum and dropping to less than 20% at 6-months postpartum; dyspareunia at 3-months postpartum was significantly more likely among women who had perineal trauma or reported pre-pregnancy dyspareunia [40].

Resumption of sex, if explored in relation to coercive or forced sex, was also included within negative sexual health outcomes. Postpartum sexual abstinence was largely practiced across settings (though length of time depended on cultural factors); however, not all women who resumed sex did so on their own accord. Specifically, in Ethiopia, among the 20% of women who had resumed sex within 6-weeks postpartum (and prior to the end of the 40 day sexual abstinence period largely observed within Ethiopia), half reported being pressured by their husband to resume intercourse [37].

While our search only uncovered three qualitative studies specific to negative sexual health, these studies were helpful for elucidating cultural beliefs and concerns surrounding sexual health in the postpartum period. In Cambodia, White explored Khmer women’s beliefs surrounding sex, specifically that resuming sex too soon after delivery, either by choice or by force, could cause physical health symptoms [42]. In Mozambique, women with fistula reported no sexual activity since onset, with one woman reporting that her husband had used her “handicap” to justify taking an additional wife [33].

Across studies/settings, many women reported sexual health morbidities following pregnancy and childbirth, however, help-seeking or participation within interventions was minimal. In Tunisia, Achour et al. reported that while women experienced vaginismus symptoms following delivery, 60% did not feel that sex was important compared to motherhood, and no women completed the pelvic floor training program nor sought counseling from the sexologist [30]. Moreover, some studies reported that women did not feel comfortable discussing sexual health issues or felt providers were poorly equipped to handle matters surrounding sexual health. In Nigeria, while 98% of women in the study reported receiving counseling on contraception, only 29% reported discussions surrounding sexual health [40]. Similarly, in Iran, women felt their sexual health needs during the postpartum period were often neglected by healthcare providers [39].

Additional findings included associations between RTIs and uterine prolapse and postpartum depression [41], a cross-sectional examination of abnormal vaginal discharges in Zambian women [38], perineal tearing and postpartum complications related to FGC in Ethiopia [35], and perineal tearing and genital prolapse in Bangladesh [34].

### Positive and negative (or neutral) sexual health

Studies that examined both positive and negative sexual health outcomes or examined women’s health within the postpartum period from a neutral perspective are outlined within Table 3 [24, 43–68] (n = 28). Of note, the majority of studies within this group explore prevalence or corelates of resumption of sex, with little discussion of the positive or negative impact of the timing of sexual activity within the postpartum period [24, 43, 46, 48–50, 53, 54, 58, 61, 66, 67]. In a multi-country DHS study, resumption of sex was related to the return of a woman’s menses [24], and this practice was corroborated via qualitative data from Cote d’Ivoire [47] and Malawi [68]. In Cote d’Ivoire, Tanzania, Eswatini, and Malawi, postpartum sexual abstinence was further described in relation to breastfeeding or child developmental benchmarks, specifically the child being of age to walk [47, 56, 63, 68].

Multiple studies linked resumption of sexual activity to their husband’s sexual needs or demands [44, 52, 55, 57, 63]. A qualitative study in Cote d’Ivoire depicted this pressure on women to resume sex—while some women felt that polygynous marriages were useful in allowing for long abstinence periods, others expressed fear of infidelity and related STI risks [47]. In one study from Kenya, increased odds of resumption of sex was associated with past-month forced sex [53].

Few studies explored specific cultural practices, withstanding timing of resumption of sex, in relation to women’s sexual health. In northern Nigeria, women reported a number of postpartum practices, including postpartum abstinence periods inclusive of confinement for 40 days after birth or longer, hot ritual baths, nursing in heated rooms, laying on heated beds, and consuming specific foods [51]. In Malawi, substantial regional variation persisted in cultural practices, however, need for postpartum abstinence was described in relation to healing the mother, partner, and child, with early resumption linked to numerous health complications [68].

Notably, within qualitative data, women described feeling less sexually attractive during the postpartum period and felt that decreased self-confidence impacted their sexual health and desire; however, they also indicated that partner acceptance of their body changes helped improve their anxiety surrounding sex [45]. Some women simply stated that they were too tired to engage in sex [54].

Within the studies on both negative and positive sexual health, women similarly reported difficulty seeking help or discussing sex with healthcare providers [44]. In one Nigerian study, fewer than two thirds of postpartum women sought help for the sexual morbidities they were experiencing, and prominent reasons for not seeking health included feeling shy, the problem resolving on its own, cultural or religious factors, and not having a female doctor to ask [52]. In Tanzania, women described that too much health education was provided at once during antenatal care, and felt that some of this information should be spread throughout postpartum care visits [56]. In Uganda, women noted that the advice provided by health workers at discharge was inconsistent, leaving them unsure of when to resume sexual activity and how to navigate associated health and safety risks [60].

## Discussion

While our search yielded 46 studies examining sexual health in the postpartum period within LMICs, only five of these studies focused exclusively on positive sexual health. Rather, sexual health was generally framed within the context of delivery complications (episiotomies, prolapse, fistula) or morbidities that continued into the postpartum period. Moreover, the vast majority of studies were conducted in sub-Saharan Africa—while this number of articles was nearly three-fold those from other geographies, many of these studies only examined the prevalence or correlates of sexual resumption. Overall, the evidence base surrounding women’s sexual health in the postpartum period within LMICs remains limited in comparison to higher income countries and highlights a need to explore a broader range of sexual health outcomes, including those surrounding positive sexual health.

This review highlights a clear need for increased sexual health education for postpartum women within LMICs. Prior global literature has highlighted a lack of education on positive sexual health, which is influenced by access to sexuality education, including knowledge of risks/vulnerabilities, access to sexual health care, and affirmative environments to promote positive sexual health [6]. Specifically, our results found that when women experienced sexual health morbidities within the postpartum period, they were hesitant to seek help from providers, both because they felt embarrassed and because they felt providers were poorly equipped to handle issues surrounding women’s sexual health. Further, many women were unclear about the recommended timing surrounding safe resumption of sex, and instead turned to cultural practices, which were largely enforced by family members. Sexual health education, including guidance surrounding care-seeking for potential sexual health morbidities that could occur, must be included within postnatal care services to ensure that women are able to resume sex when they feel the time is right for themselves and their relationships. Additionally, normalization of sexual health dysfunction, particularly at a time when women’s bodies are undergoing massive physical and hormonal changes, can help women feel less embarrassed and stigmatized. Clear guidelines for healthcare providers on how to integrate sexual health education into postpartum protocols are necessary, alongside the expansion of accessible postpartum care services far into the postpartum period given delays in resumption of sexual activity and recognition of associated dysfunctions.

While maternal health services are rapidly expanding within LMICs, attention is shifting to not only ensure provision of services, but to also assess the quality of such services [16]. Current quality of care assessments, however, do not include sexual health counseling and to date, the majority have focused on content of antenatal services, with little attention to quality of postnatal care services [69]. Without data to examine receipt and content of sexual health education and counseling as part of antenatal and/or postnatal care services, the research and practice field does not have a clear sense of when and how sexual health is addressed. Future work must seek to close this data gap given the profound impact these data will have on shaping clinical practice guidelines and in turn, monitoring quality of care.

Strengths of this study include a rigorously implemented scoping protocol, utilization of library informationists in developing and revising the search strategy, and focus on an understudied topic inclusive of both positive and negative sexual health outcomes; however, this study is not without limitations. Namely, this scoping review includes many studies with small samples or limited measures for sexual health in the postpartum period. The majority of studies included were cross-sectional in nature, limiting conclusions surrounding temporality of associations. Further, some studies did not define inclusion criteria surrounding timing within postpartum period, and thus were not included within the current scoping review as we could not assess whether the findings were specific to one year postpartum. Methodologic rigor was not assessed as part of this review given the large number of articles retained within our final criteria. Lastly, as our search strategy purposively did not examine women’s sexual health in relation to fertility and contraception, it is possible that some articles which also explored women’s sexual health in the postpartum period more broadly may have been missed by our search.

## Conclusion

In conclusion, we urge future research to examine sexual health beyond the resumption of sex after childbirth and to explore barriers to help-seeking for women experiencing sexual health morbidities in the postpartum period. Further exploration of positive women’s sexual health is needed, inclusive of factors that may promote positive sexual health within the postpartum period. Ultimately, both the expansion of indicators surrounding positive sexual health and prioritization of women’s sexual health within global development priorities, such as the SDGs, can ensure that the full range of women’s health needs are not only addressed, but also valued, regardless of context.

## Supplementary Information

**Additional file 1: Table S1.** PubMed MeSH Terms

## Data Availability

Original articles reviewed in this manuscript are available on PubMed.

## Abbreviations

DHS: Demographic and Health Surveys
FGC: Female genital cutting
FSFI: Female Sexual Function Index
HIV: Human immunodeficiency virus
LMIC: Low- and middle-income country
RTI: Reproductive tract infection
SRH: Sexual and reproductive health
STI: Sexually transmitted infection
