# Supplementary material for: A scoping review on women’s sexual health in the postpartum period: opportunities for research and practice within low-and middle-income countries
Source: Reprod Health. 2022 May 8;19:112. doi: 10.1186/s12978-022-01399-6 (PMC9079206; doi:10.1186/s12978-022-01399-6)
Supplement: Supplementary file 1 — Additional file 1: Table S1. PubMed MeSH Terms [file 12978_2022_1399_MOESM1_ESM.docx]

**Supplementary Table 1. PubMed MeSH Terms**

| ((((("Sexuality"[Mesh] AND (1000/1/1:2017/12/31[pdat])) OR "Sexual Health"[Mesh] OR "Circumcision, Female"[Mesh] OR "Sex Offenses"[Mesh] OR "Sexually Transmitted Diseases"[Mesh] OR "Vaginal Fistula"[Mesh] OR "Uterine Prolapse"[Mesh] OR "genital diseases, female"[Mesh] OR "Sexual Dysfunctions, Psychological"[Mesh] OR "Orgasm"[Mesh] OR "Sexual Arousal"[Mesh] OR Coitus[MeSH] OR "sexual health"[tiab] OR Sexuality[tiab] OR "women’s health"[tiab] OR (Harm*[tiab] AND (“cultural practice*”[tiab] OR “traditional practice*”[tiab] OR “traditional belief*”[tiab])) OR "sexually transmitted infection"[tiab] OR "sexually transmitted infections"[tiab] OR STI[tiab] OR STIs[tiab] OR "sexually transmitted disease"[tiab] OR "sexually transmitted diseases"[tiab] OR STD[tiab] OR STDs[tiab] OR "venereal disease"[tiab] OR "venereal diseases"[tiab] OR "reproductive tract infection"[tiab] OR "reproductive tract infections"[tiab] OR RTI[tiab] OR RTIs[tiab] OR "sexual dysfunction"[tiab] OR "sexual disorder"[tiab] OR "sexual disorders"[tiab] OR "sex disorder"[tiab] OR "sex disorders"[tiab] OR "desire disorder"[tiab] OR "desire disorders"[tiab] OR "arousal disorder"[tiab] OR "arousal disorders"[tiab] OR "orgasm disorder"[tiab] OR "orgasm disorders"[tiab] OR vaginismus[tiab] OR vulvodynia[tiab] OR dyspareunia[tiab] OR "sex offense"[tiab] OR "sex offenses"[tiab] OR "sexual offense"[tiab] OR "sexual offenses"[tiab] OR "sex offence"[tiab] OR "sex offences"[tiab] OR "sexual offence"[tiab] OR "sexual offences"[tiab] OR "sexual violence"[tiab] OR "sexual violences"[tiab] OR "sexual abuse"[tiab] OR "sexual abuses"[tiab] OR "sexual assault"[tiab] OR "sexual assaults"[tiab] OR rape[tiab] OR "sexual coercion"[tiab] OR "female genital cutting"[tiab] OR FGC[tiab] OR "female genital mutilation"[tiab] OR FGM[tiab] OR FGC/FGM[tiab] OR "female circumcision"[tiab] OR ("female"[tiab] AND "circumcisions"[tiab]) OR infibulation[tiab] OR infibulations[tiab] OR clitoridectomy[tiab] OR clitoridectomies[tiab] OR clitorectomy[tiab] OR clitorectomies[tiab] OR fistula[tiab] OR fistulas[tiab] OR prolapse[tiab] OR prolapses[tiab] OR "sexual function"[tiab] OR "sexual pleasure"[tiab] OR "sexual satisfaction"[tiab] OR orgasm[tiab] OR orgasms[tiab] OR "sexual gratification"[tiab] OR ("sexual"[tiab] AND "gratifications"[tiab]) OR arousal[tiab] OR "sexual excitement"[tiab] OR intimacy[tiab] OR abstinence[tiab] OR "sexual activity"[tiab] OR intercourse[tiab])  AND  ("Postpartum Period"[Mesh] OR Puerperium[Mesh] OR "postpartum period*" [tiab] OR Puerperium[tw]))  AND  (((((afghanistan[MeSH] OR albania[MeSH] OR algeria[MeSH] OR american samoa[MeSH] OR angola[MeSH] OR antigua and barbuda[MeSH] OR argentina[MeSH] OR armenia[MeSH] OR aruba[MeSH] OR azerbaijan[MeSH] OR bahrain[MeSH] OR bangladesh[MeSH] OR barbados[MeSH] OR republic of belarus[MeSH] OR belize[MeSH] OR benin[MeSH] OR bhutan[MeSH] OR bolivia[MeSH] OR bosnia and herzegovina[MeSH] OR botswana[MeSH] OR brazil[MeSH] OR bulgaria[MeSH] OR burkina faso[MeSH] OR burundi[MeSH] OR cabo verde[MeSH] OR cambodia[MeSH] OR cameroon[MeSH] OR central african republic[MeSH] OR chad[MeSH] OR chile[MeSH] OR china[MeSH] OR colombia[MeSH] OR comoros[MeSH] OR democratic republic of the congo[MeSH] OR congo[MeSH] OR costa rica[MeSH] OR cote d’ivoire[MeSH] OR croatia[MeSH] OR cuba[MeSH] OR cyprus[MeSH] OR czech republic[MeSH] OR djibouti[MeSH] OR dominica[MeSH] OR dominican republic[MeSH] OR ecuador[MeSH] OR egypt[MeSH] OR el salvador[MeSH] OR equatorial guinea[MeSH] OR eritrea[MeSH] OR estonia[MeSH] OR eswatini[Mesh] OR swaziland[MeSH] OR ethiopia[MeSH] OR fiji[MeSH] OR gabon[MeSH] OR gambia[MeSH] OR "georgia (republic)"[MeSH] OR ghana[MeSH] OR gibraltar[MeSH] OR grenada[MeSH] OR guam[MeSH] OR guatemala[MeSH] OR guinea[MeSH] OR guinea bissau[MeSH] OR guyana[MeSH] OR haiti[MeSH] OR honduras[MeSH] OR india[MeSH] OR indonesia[MeSH] OR iran[MeSH] OR iraq[MeSH] OR jamaica[MeSH] OR jordan[MeSH] OR kazakhstan[MeSH] OR kenya[MeSH] OR "democratic people’s republic of korea"[MeSH] OR republic of korea[MeSH] OR kosovo[MeSH] OR kyrgyzstan[MeSH] OR laos[MeSH] OR latvia[MeSH] OR lebanon[MeSH] OR lesotho[MeSH] OR liberia[MeSH] OR libya[MeSH] OR lithuania[MeSH] OR macau[MeSH] OR "republic of north macedonia"[MeSH] OR madagascar[MeSH] OR malawi[MeSH] OR malaysia[MeSH] OR indian ocean islands[MeSH] OR mali[MeSH] OR malta[MeSH] OR micronesia[MeSH] OR palau[MeSH] OR mauritania[MeSH] OR mauritius[MeSH] OR mexico[MeSH] OR moldova[MeSH] OR mongolia[MeSH] OR montenegro[MeSH] OR morocco[MeSH] OR mozambique[MeSH] OR myanmar[MeSH] OR namibia[MeSH] OR nepal[MeSH] OR netherlands antilles[MeSH] OR nicaragua[MeSH] OR niger[MeSH] OR nigeria[MeSH] OR oman[MeSH] OR pakistan[MeSH] OR panama[MeSH] OR papua new guinea[MeSH] OR paraguay[MeSH] OR peru[MeSH] OR philippines[MeSH] OR poland[MeSH] OR portugal[MeSH] OR puerto rico[MeSH] OR romania[MeSH] OR russia[MeSH] OR rwanda[MeSH] OR samoa[MeSH] OR sao tome and principe[MeSH] OR saudi arabia[MeSH] OR senegal[MeSH] OR serbia[MeSH] OR seychelles[MeSH] OR sierra leone[MeSH] OR slovakia[MeSH] OR slovenia[MeSH] OR melanesia[MeSH] OR somalia[MeSH] OR south africa[MeSH] OR south sudan[MeSH] OR sri lanka[MeSH] OR saint kitts and nevis[MeSH] OR saint lucia[MeSH] OR saint vincent and the grenadines[MeSH] OR sudan[MeSH] OR suriname[MeSH] OR syria[MeSH] OR tajikistan[MeSH] OR tanzania[MeSH] OR thailand[MeSH] OR timor leste[MeSH] OR togo[MeSH] OR tonga[MeSH] OR trinidad and tobago[MeSH] OR tunisia[MeSH] OR "turkey"[MeSH] OR turkmenistan[MeSH] OR uganda[MeSH] OR ukraine[MeSH] OR uruguay[MeSH] OR uzbekistan[MeSH] OR vanuatu[MeSH] OR venezuela[MeSH] OR vietnam[MeSH] OR middle east[MeSH] OR yemen[MeSH] OR yugoslavia[MeSH] OR zambia[MeSH] OR zimbabwe[MeSH] OR africa south of the sahara[MeSH] OR africa, central[MeSH] OR africa, northern[MeSH] OR africa, southern[MeSH] OR africa, eastern[MeSH] OR africa, western[MeSH] OR west indies[MeSH] OR indian ocean islands[MeSH] OR caribbean region[MeSH] OR central america[MeSH] OR latin america[MeSH] OR south america[MeSH] OR asia, central[MeSH] OR asia, northern[MeSH] OR asia, southeastern[MeSH] OR asia, western[MeSH] OR europe, eastern[MeSH] OR developing countries[MeSH]) OR (afghanistan[Text Word] OR albania[Text Word] OR algeria[Text Word] OR american samoa[Text Word] OR angola[Text Word] OR antigua[Text Word] OR barbuda[Text Word] OR argentina[Text Word] OR armenia[Text Word] OR armenian[Text Word] OR aruba[Text Word] OR azerbaijan[Text Word] OR bahrain[Text Word] OR bangladesh[Text Word] OR barbados[Text Word] OR belarus[Text Word] OR byelarus[Text Word] OR belorussia[Text Word] OR byelorussian[Text Word] OR belize[Text Word] OR british honduras[Text Word] OR benin[Text Word] OR dahomey[Text Word] OR bhutan[Text Word] OR bolivia[Text Word] OR bosnia[Text Word] OR herzegovina[Text Word] OR botswana[Text Word] OR bechuanaland[Text Word] OR brazil[Text Word] OR brasil[Text Word] OR bulgaria[Text Word] OR burkina faso[Text Word] OR burkina fasso[Text Word] OR upper volta[Text Word] OR burundi[Text Word] OR urundi[Text Word] OR cabo verde[Text Word] OR cape verde[Text Word] OR cambodia[Text Word] OR kampuchea[Text Word] OR khmer republic[Text Word] OR cameroon[Text Word] OR cameron[Text Word] OR cameroun[Text Word] OR central african republic[Text Word] OR ubangi shari[Text Word] OR chad[Text Word] OR chile[Text Word] OR china[Text Word] OR colombia[Text Word] OR comoros[Text Word] OR comoro islands[Text Word] OR mayotte[Text Word] OR congo[Text Word] OR zaire[Text Word] OR costa rica[Text Word] OR cote d'ivoire[Text Word] OR cote d'ivoire[Text Word] OR cote d'ivoire[Text Word] OR ivory coast[Text Word] OR croatia[Text Word] OR cuba[Text Word] OR cyprus[Text Word] OR czech republic[Text Word] OR czechoslovakia[Text Word] OR djibouti[Text Word] OR french somaliland[Text Word] OR dominica[Text Word] OR dominican republic[Text Word] OR ecuador[Text Word] OR egypt[Text Word] OR united arab republic[Text Word] OR el salvador[Text Word] OR equatorial guinea[Text Word] OR spanish guinea[Text Word] OR eritrea[Text Word] OR estonia[Text Word] OR eswatini[Text Word] OR swaziland[Text Word] OR ethiopia[Text Word] OR fiji[Text Word] OR gabon[Text Word] OR gabonese republic[Text Word] OR gambia[Text Word] OR georgia[Text Word] OR georgian[Text Word] OR ghana[Text Word] OR gold coast[Text Word] OR gibraltar[Text Word] OR grenada[Text Word] OR guam[Text Word] OR guatemala[Text Word] OR guinea[Text Word] OR guyana[Text Word] OR guiana[Text Word] OR haiti[Text Word] OR hispaniola[Text Word] OR honduras[Text Word] OR india[Text Word] OR indonesia[Text Word] OR timor[Text Word] OR iran[Text Word] OR iraq[Text Word] OR isle of man[Text Word] OR jamaica[Text Word] OR jordan[Text Word] OR kazakhstan[Text Word] OR kazakh[Text Word] OR kenya[Text Word] OR korea[Text Word] OR kosovo[Text Word] OR kyrgyzstan[Text Word] OR kirghizia[Text Word] OR kirgizstan[Text Word] OR kyrgyz republic[Text Word] OR kirghiz[Text Word] OR laos[Text Word] OR lao pdr[Text Word] OR lao people's democratic republic[Text Word] OR latvia[Text Word] OR lebanon[Text Word] OR lesotho[Text Word] OR basutoland[Text Word] OR liberia[Text Word] OR libya[Text Word] OR libyan arab jamahiriya[Text Word] OR lithuania[Text Word] OR macau[Text Word] OR macao[Text Word] OR macedonia[Text Word] OR madagascar[Text Word] OR malagasy republic[Text Word] OR malawi[Text Word] OR nyasaland[Text Word] OR malaysia[Text Word] OR maldives[Text Word] OR indian ocean[Text Word] OR mali[Text Word] OR malta[Text Word] OR micronesia[Text Word] OR kiribati[Text Word] OR marshall islands[Text Word] OR nauru[Text Word] OR northern mariana islands[Text Word] OR palau[Text Word] OR tuvalu[Text Word] OR mauritania[Text Word] OR mauritius[Text Word] OR mexico[Text Word] OR moldova[Text Word] OR moldovian[Text Word] OR mongolia[Text Word] OR montenegro[Text Word] OR morocco[Text Word] OR ifni[Text Word] OR mozambique[Text Word] OR portuguese east africa[Text Word] OR myanmar[Text Word] OR burma[Text Word] OR namibia[Text Word] OR nepal[Text Word] OR netherlands antilles[Text Word] OR nicaragua[Text Word] OR niger[Text Word] OR nigeria[Text Word] OR oman[Text Word] OR muscat[Text Word] OR pakistan[Text Word] OR panama[Text Word] OR papua new guinea[Text Word] OR paraguay[Text Word] OR peru[Text Word] OR philippines[Text Word] OR philipines[Text Word] OR phillipines[Text Word] OR phillippines[Text Word] OR poland[Text Word] OR polish people's republic[Text Word] OR portugal[Text Word] OR portuguese republic[Text Word] OR puerto rico[Text Word] OR romania[Text Word] OR russia[Text Word] OR russian federation[Text Word] OR ussr[Text Word] OR soviet union[Text Word] OR union of soviet socialist republics[Text Word] OR rwanda[Text Word] OR ruanda[Text Word] OR samoa[Text Word] OR pacific islands[Text Word] OR polynesia[Text Word] OR samoan islands[Text Word] OR sao tome and principe[Text Word] OR saudi arabia[Text Word] OR senegal[Text Word] OR serbia[Text Word] OR seychelles[Text Word] OR sierra leone[Text Word] OR slovakia[Text Word] OR slovak republic[Text Word] OR slovenia[Text Word] OR melanesia[Text Word] OR solomon island[Text Word] OR solomon islands[Text Word] OR norfolk island[Text Word] OR somalia[Text Word] OR south africa[Text Word] OR south sudan[Text Word] OR sri lanka[Text Word] OR ceylon[Text Word] OR saint kitts and nevis[Text Word] OR st kitts and nevis[Text Word] OR saint lucia[Text Word] OR st lucia[Text Word] OR saint vincent[Text Word] OR st vincent[Text Word] OR grenadines[Text Word] OR sudan[Text Word] OR suriname[Text Word] OR surinam[Text Word] OR syria[Text Word] OR syrian arab republic[Text Word] OR tajikistan[Text Word] OR tadjikistan[Text Word] OR tadzhikistan[Text Word] OR tadzhik[Text Word] OR tanzania[Text Word] OR tanganyika[Text Word] OR thailand[Text Word] OR siam[Text Word] OR timor leste[Text Word] OR east timor[Text Word] OR togo[Text Word] OR togolese republic[Text Word] OR tonga[Text Word] OR trinidad[Text Word] OR tobago[Text Word] OR tunisia[Text Word] OR turkey[Text Word] OR turkmenistan[Text Word] OR turkmen[Text Word] OR uganda[Text Word] OR ukraine[Text Word] OR uruguay[Text Word] OR uzbekistan[Text Word] OR uzbek[Text Word] OR vanuatu[Text Word] OR new hebrides[Text Word] OR venezuela[Text Word] OR vietnam[Text Word] OR viet nam[Text Word] OR middle east[Text Word] OR west bank[Text Word] OR gaza[Text Word] OR palestine[Text Word] OR yemen[Text Word] OR yugoslavia[Text Word] OR zambia[Text Word] OR zimbabwe[Text Word] OR northern rhodesia[Text Word] OR global south[Text Word] OR africa south of the sahara[Text Word] OR sub saharan africa[Text Word] OR subsaharan africa[Text Word] OR central africa[Text Word] OR north africa[Text Word] OR northern africa[Text Word] OR magreb[Text Word] OR maghrib[Text Word] OR sahara[Text Word] OR southern africa[Text Word] OR east africa[Text Word] OR eastern africa[Text Word] OR west africa[Text Word] OR western africa[Text Word] OR west indies[Text Word] OR indian ocean islands[Text Word] OR caribbean[Text Word] OR central america[Text Word] OR latin america[Text Word] OR south america[Text Word] OR central asia[Text Word] OR north asia[Text Word] OR northern asia[Text Word] OR southeastern asia[Text Word] OR south eastern asia[Text Word] OR southeast asia[Text Word] OR south east asia[Text Word] OR western asia[Text Word] OR east europe[Text Word] OR eastern europe[Text Word] OR developing country[Text Word] OR developing countries[Text Word] OR developing nation[Text Word] OR developing nations[Text Word] OR developing population[Text Word] OR developing populations[Text Word] OR developing world[Text Word] OR less developed country[Text Word] OR less developed countries[Text Word] OR less developed nation[Text Word] OR less developed nations[Text Word] OR less developed world[Text Word] OR lesser developed countries[Text Word] OR lesser developed nations[Text Word] OR under developed country[Text Word] OR under developed countries[Text Word] OR under developed nations[Text Word] OR under developed world[Text Word] OR underdeveloped country[Text Word] OR underdeveloped countries[Text Word] OR underdeveloped nation[Text Word] OR underdeveloped nations[Text Word] OR underdeveloped population[Text Word] OR underdeveloped populations[Text Word] OR underdeveloped world[Text Word] OR middle income country[Text Word] OR middle income countries[Text Word] OR middle income nation[Text Word] OR middle income nations[Text Word] OR middle income population[Text Word] OR middle income populations[Text Word] OR low income country[Text Word] OR low income countries[Text Word] OR low income nation[Text Word] OR low income nations[Text Word] OR low income population[Text Word] OR low income populations[Text Word] OR lower income country[Text Word] OR lower income countries[Text Word] OR lower income nations[Text Word] OR lower income population[Text Word] OR lower income populations[Text Word] OR underserved countries[Text Word] OR underserved nations[Text Word] OR underserved population[Text Word] OR underserved populations[Text Word] OR under served population[Text Word] OR under served populations[Text Word] OR deprived countries[Text Word] OR deprived population[Text Word] OR deprived populations[Text Word] OR poor country[Text Word] OR poor countries[Text Word] OR poor nation[Text Word] OR poor nations[Text Word] OR poor population[Text Word] OR poor populations[Text Word] OR poor world[Text Word] OR poorer countries[Text Word] OR poorer nations[Text Word] OR poorer population[Text Word] OR poorer populations[Text Word] OR developing economy[Text Word] OR developing economies[Text Word] OR less developed economy[Text Word] OR less developed economies[Text Word] OR underdeveloped economies[Text Word] OR middle income economy[Text Word] OR middle income economies[Text Word] OR low income economy[Text Word] OR low income economies[Text Word] OR lower income economies[Text Word] OR low gdp[Text Word] OR low gnp[Text Word] OR low gross domestic[Text Word] OR low gross national[Text Word] OR lower gdp[Text Word] OR lower gross domestic[Text Word] OR lmic[Text Word] OR lmics[Text Word] OR third world[Text Word] OR lami country[Text Word] OR lami countries[Text Word] OR transitional country[Text Word] OR transitional countries[Text Word] OR emerging economies[Text Word] OR emerging nation[Text Word] OR emerging nations[Text Word]))))))  NOT ("Infectious Disease Transmission, Vertical"[Mesh]))  NOT (animals [mesh] NOT humans[mesh]) |
| --- |
